# Acute exercise impacts heart rate variability but not cognitive flexibility during subsequent simulated firefighter occupational tasks

**DOI:** 10.1007/s00421-024-05650-9

**Published:** 2024-11-13

**Authors:** Philip J. Agostinelli, Nicholas C. Bordonie, Braxton A. Linder, Ann M. Robbins, Parker L. Jones, Lee F. Reagan, C. Brooks Mobley, Matthew W. Miller, William M. Murrah, JoEllen M. Sefton

**Affiliations:** 1 Warrior Research Center, School of Kinesiology, Auburn University, 301 Wire Road, Auburn, AL 38632 USA; 2 Neurovascular Physiology Lab, Auburn University, Auburn, AL USA; 3 Nutrabolt Applied and Molecular Physiology Lab, Auburn University, Auburn, AL USA; 4 Performance and Exercise Psychophysiology Lab, Auburn University, Auburn, AL USA; 5 Department of Educational Foundations, Leadership, and Technology, Auburn University, Auburn, AL USA

**Keywords:** Executive function, Tactical athletes, Heart rate variability, Heat stress, High-intensity interval training, Resistance training

## Abstract

**Purpose:**

Acute exercise can transiently enhance cognitive flexibility. The cognitive demand of firefighters makes it relevant to understand if on-shift exercise could produce similar improvements in cognitive performance during subsequent occupational tasks. Metrics of heart rate variability (HRV), such as time- and frequency-domain outcomes, may shed light upon the influence exercise has on cognition, as they discern information related to cardiac autonomic (sympathetic/parasympathetic) function. We aimed to determine if acute resistance and aerobic exercise impact cognitive flexibility during occupational tasks and its relation to HRV.

**Methods:**

32 participants completed a baseline Wisconsin Card Sorting Task (WCST) and three experimental trials: resistance exercise (RE), aerobic exercise (AE), or a rested control (CON). An occupational task assessment (OTA) including four rounds of 10 deadlifts and a 0.15-mile sandbag carry in an environmental chamber (35 °C/50% humidity) was completed after each trial. The second round was followed by the WCST. Repeated measures ANOVAs were used to analyze differences by condition.

**Results:**

For the WCST, total, perseverative, and non-perseverative errors did not differ (ps > 0.39). Time-domain HRV metrics were not different (ps > 0.05). All frequency-domain metrics, other than low-frequency power, were not different (ps > 0.24). Low-frequency power was lower based on condition (p = 0.03). Post hoc analysis showed low-frequency power was lower following AE compared to RE and CON.

**Conclusion:**

Results suggest an acute bout of on-shift aerobic or resistance exercise may not impact cognitive flexibility during subsequent simulated occupational tasks, despite depressed metrics of heart rate variability following aerobic exercise.

## Introduction

In addition to the obvious physical demands of firefighters, cognitive function is a vital component of their occupational responsibilities (Kujawski et al. 2018; Kwak et al. 2020). These occupational demands require high levels of executive function to make rapid life-or-death decisions in challenging situations and environments (Coehoorn et al. 2020; Sanfey 2007). Executive function refers to the grouping of effortful mental processes necessary for attention and decision-making (Diamond 2013). Cognitive flexibility, a subdomain of executive function, is the ability to utilize both inhibitory control and working memory to make quick, situational decisions. This ability to make situational decisions, suggest cognitive flexibility is a valuable component of occupational readiness in firefighters (Kujawski et al. 2018; Kwak et al. 2020).

Heart rate variability (HRV) is one potential factor that provides insight into the connection between exertion, physiology, and cognitive function. HRV is the measure of variation in the time interval between heartbeats (Jimenez Morgan & Molina Mora 2017; Thayer et al. 2010) and represents the body’s ability to adjust to changing demands through variations in parasympathetic and sympathetic activities (Capilupi et al. 2020; Hewett et al. 2017; Thayer et al. 2010). HRV decreases in response to an acute bout of exercise due to the rise in sympathetic activity needed to increase the cardiac output and systemic vascular tone required to meet physiological demands of exercise (Michael et al. 2017, 2016; Yılmaz et al. 2018). It has been proposed that neuroanatomical connections between the central and autonomic nervous systems provide an interaction between parasympathetic cardiac autonomic regulation and executive function controlled by the prefrontal cortex (Thayer et al. 2012). Various studies have reported individuals who performed better in cognitive tasks often have greater cardiac parasympathetic control than those with poorer cognitive performance (Hansen et al. 2003; Kimhy et al. 2013; Luque-Casado et al. 2016; Thayer et al. 2012). Consideration of physical and cognitive function in firefighters is important for the maintenance of occupational readiness.

Research has shown that executive functions (working memory and inhibitory control) and other cognitive functions (information processing and psychomotor control) (Kwak et al. 2020; Morley et al. 2012; Sumińska et al. 2021; Zare et al. 2018) decline during simulated firefighting. One potential method to mitigate these declines is on-shift exercise. Research indicates cognitive flexibility is transiently improved following an acute bout of aerobic exercise (Bae & Masaki 2019; Dupuy et al. 2018; Dwojaczny et al. 2020), however the acute effects of resistance exercise on cognitive performance have been less widely studied. Research suggests components of executive function such as inhibitory control are improved following acute resistance exercise, but the effect on cognitive flexibility and working memory remain unclear (Soga et al. 2018).

While evidence suggests that a single on-shift exercise session may transiently impair a firefighter’s ability to meet occupational demands (Dennison et al. 2012; Mason et al. 2023; Smith et al. 2001), there may be a cognitive benefit to acute on-shift exercise. However, no study to our knowledge has directly compared the acute impact of resistance training and aerobic endurance training on cognitive readiness in firefighters and its relation to HRV. Therefore, the aim of this study was to investigate the impact of resistance and aerobic endurance exercise on cognitive outcomes related to firefighter occupational performance and the influence of HRV on cognitive performance. We hypothesized that prior-aerobic exercise will result in improvement in cognitive flexibility and smaller decreases in HRV metrics, but prior resistance exercise will result in lower cognitive flexibility and larger decreases HRV metrics (Heffernan et al. 2006) during a cognitive occupational task assessment, when compared to a rested control condition.

## Methods

### Study participants

**Fig. 1:**
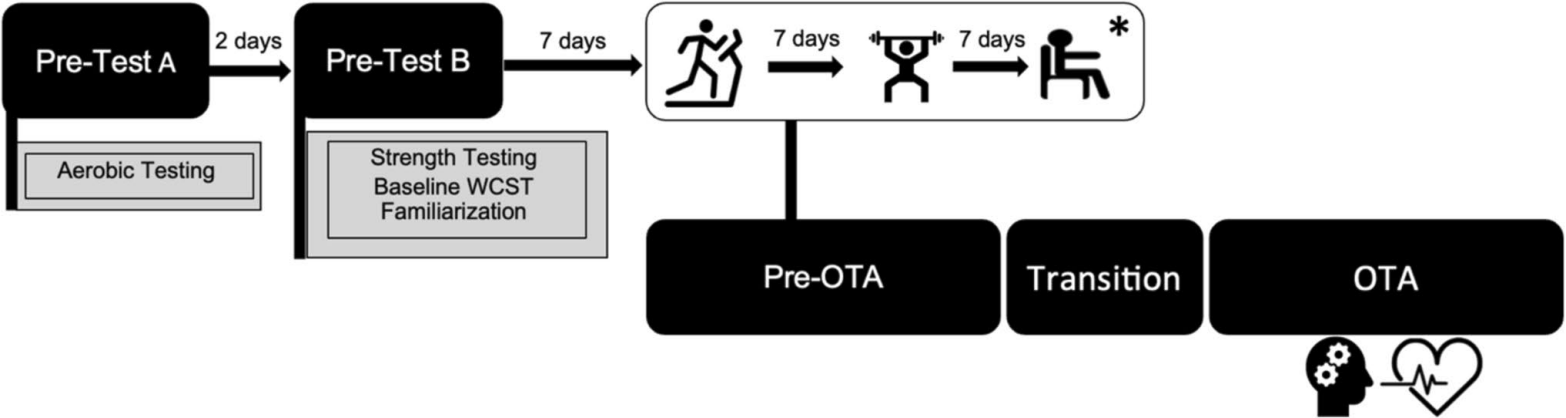
Aerobic testing included VO_2_peak and lactate threshold assessments; strength testing included 3RM maximum lifts for bench, squat, and deadlift; WCST: Wisconsin Card Sorting Task; familiarization involved exposure to the occupational task assessment (OTA) and environmental conditions; *Pre-OTA exercise condition (aerobic, resistance, or rest) order was randomized. Transition was the time used to replicate response time and involved donning firefighter gear and walking to the environmental chamber. The WCST was completed halfway through the OTA. Heart rate variability was monitored continuously during the WCST

Data herein are part of a larger study approved by the Institutional Review Board at Auburn University (IRB protocol code #22-479 AR 2211). Of 41 participants recruited, 29 completed study requirements for the current investigation. These participants included 13 females and 16 males from the local community (not active firefighters) that met the following criteria: ages 19–45 years old, no current musculoskeletal injury, available to complete all study visits, comfortable exercising in an environmental chamber, agreed to adhere to study requirements, and passed a physical activity readiness questionnaire (PAR-Q) health screening. Exclusion criteria included known medical condition, physical or psychological condition preventing participation in exercise, diagnosed with asthma in the past 4 years, or recently experienced a heat-injury. Informed consent was obtained prior to enrollment in the study. A schematic of the study design can be found in Fig. 1**.**

### Research design

This manuscript reports a secondary analysis of variables measured during a repeated-measures quasi-randomized control study (Agostinelli et al. 2024). The quasi-randomization involved randomizing which acute exercise modality was completed first (AE or RE) with all participants completing the CON condition second. This was in effort to reduce the number of potential unique trial order group comparisons. Block randomization of exercise trial order was generated using Microsoft^®^ Excel (Microsoft Corporations, Redmond, WA USA) by a co-investigator and allocations were reported to the lead author (PA). Participants completed a total of five visits, the first two visits including a baseline assessment for cognitive function, anaerobic fitness, and aerobic fitness. Participants then completed three experimental conditions: (1) resistance exercise (RE), (2) aerobic high-intensity interval training (AE), and (3) rested control (CON); each followed immediately with an occupational task assessment (OTA). A 7-day wash-out period was included between trials to reduce the risk of residual fatigue impacting subsequent trial performance. Randomization details have been previously submitted for publication (Agostinelli et al. 2024).

### Wisconsin card sorting task (WCST) and resting heart rate

Participants first completed a 10-min resting period to assess resting heart rate for heart rate reserve (HRR) estimation for the aerobic exercise intensity prescription. A baseline assessment of cognitive flexibility was completed using the modified Wisconsin Card Sorting Task (WCST) to control for the practice effect associated with the task prior to completing all physically demanding baseline assessments (Basso et al. 2001). The modified WCST required participants to complete 60 total responses, instead of the traditional 60 correct responses with up to 128 total trials (Miles et al. 2021), for the sake of time efficiency during the OTA. The WCST uses a computer-based card sorting task completed in a quiet space with no potential distractors (devices, flashing lights, clocks, posters, etc.). During acute exercise trials the WCST was completed halfway through the occupational task assessment (OTA) while still in the active environmental chamber with no external noise or potential distractors. Participants sorted the cards based on three factors: color, shape, and number. The category (the sorting rule) changes after sorting 10 cards. This was repeated for a total of six sorting categories (60 total responses). While not a direct sensitivity or reliability analysis has been done on this version of the WCST, previous literature supports the sensitivity, reliability, and clinical use of shortened computerized modified-WCSTs (Greve 2001; Schretlen 2010; Steinke et al. 2021). Our primary variable was perseverative errors. These errors represent a participant’s inability to adapt to the new sorting rule and are representative of task-switching and cognitive flexibility (Landry & Mitchell 2021). Additional secondary measures include response time, correct responses, errors, and non-perseverative errors (Kohli & Kaur 2006). Participants were prompted to complete the assessment as fast as possible while minimizing errors. Instructions outlined on the computer screen reviewed the rules of the WCST before beginning the assessment to reduce the variability of instructions from individual researchers.

### Baseline fitness testing

After the baseline WCST was completed three-repetition maximum testing (3RM) performance for barbell bench, squat, and hex-bar deadlift occurred. Participants started the 3RM protocol by completing the lift with a series of warm-up lifts (45-lb bar for 8–10 reps, 50% of projected 1-RM for 6–8 reps, 75% for 3–5 reps, and 85% for 1–3 reps). Their first attempt was at 95% of self-reported 3RM. Each set included a minimum of 2 min of rest and a maximum of 5 min of rest. The maximum weight completed for three reps while maintaining good form was utilized to project 1RM based on the National Strength and Conditioning Association’s (NSCA) guidelines (Landers 1984). If participants only achieved one or two reps at attempted weight, that value was taken and utilized to project 1RM. These assessments were used to determine training load during the acute resistance training session. Additional information related to the protocol used to obtain the participants 3RM has previously submitted for publication (Agostinelli et al. 2024). Participants were instructed on how to don standard firefighter personal protective equipment (PPE), including turn-out gear, gloves, boots, and helmet. They then entered the environmental chamber set at 35 °C (95°F) with 50% relative humidity and practiced one round of the OTA to reduce any learning effect during testing. Additionally on the second baseline testing visit peak oxygen consumption (VO_2_peak), lactate threshold, and max heart rate were collected utilizing a treadmill (Woodway, Waukesha, WI, USA) and the modified-Bruce protocol (Trabulo et al. 1994) while monitoring oxygen consumption and carbon dioxide indirect calorimetry via an automated open circuit system (Parvo Medics, Sandy UT). Max heart rate achieved during the assessment was utilized to calculated HRR for the acute aerobic exercise prescription. Oxygen consumption and lactate threshold measures unrelated to the current analysis have been submitted for publication previously (Agostinelli et al. 2024).

### Acute exercise and control sessions

Participants were asked to refrain from caffeine and exercise during 24 h leading up to each visit. Urine specific gravity (USG) was assessed to determine if participants were adequately hydrated prior to completing the session (USG < 1.025). Those who did not meet this USG value were given water and reassessed prior to starting the visit. The RE condition included a full body resistance workout (Table 1) following National Strength and Conditioning Association (NSCA) guidelines for strength training (Sheppard & Triplett 2016). If the prescribed weight was too heavy for the participant, the weight was lowered to finish the set and all remaining sets. The AE session (Table 2) involved high-intensity interval training (HIIT) on a treadmill (Woodway, Waukesha, WI, USA). The HRR values were calculated with the Karvonen method (Karvonen et al. 1957). Exercise sessions were designed based on consideration of commonly accessible equipment for firefighters in the southeastern United States (Agostinelli et al. 2023) as well as the local fire departments insight on common exercise routines of their firefighters. A control session was completed to assess any effect of exercise in general, regardless of modality. During the control session participants were seated in a quiet, controlled environment. This session was 30 min in duration and participants were instructed to engage in activities they would normally do with 30 min of down time (i.e., working on laptops, answering emails, scrolling on their phone, or reading a book), but to avoid stressful/overstimulating activities. After all sessions, Participants then transitioned to the environmental chamber and put on standard issue firefighter gear including a helmet, bunker jacket and pants, gloves, and boots.

**Table 1:** Resistance exercise session prescription

Exercise	Weight	RPE	Sets	Reps
Barbell back squat	80% 1RM	8	4	6
Barbell bench	80% 1RM	8	4	6
Hex-bar deadlift	80% 1RM	8	4	6
Barbell bent-over row	*	8	4	6

*1RM* 1 repetition maximum lift, *RPE* rating of perceived exertion, *Reps* repetitions,

^*^prescribed weight for the barbell bent-over row weight was estimated based on RPE during warm-up sets

**Table 2:** Aerobic exercise session prescription

Exercise	Duration	Intensity	Rounds
Low-intensity intervals	4 min	50% of HRR	4
High-intensity intervals	30 s	90% of HRR	4

*HRR* heart rate reserve [(max heart rate – resting heart rate)/resting heart rate)]

### Occupational task assessment (OTA)

Participants then quickly transitioned from acute exercise or rest to the environmental chamber. The environmental conditions were set to 35 °C (95°F) and 50% humidity. This was chosen based on the prevalence of these conditions during firefighter emergency calls in the southeastern United States (Agostinelli et al. 2023; Aisbett et al. 2012; Williams-Bell et al. 2017). These conditions represent a hot summer day, outdoor emergencies, and salvage rather than structural fire conditions. The transition period was not controlled, but rather participants were encouraged to respond as quickly as possible. This was an intentional decision rather than standardizing the transition period, to reduce potential recovery from passive rest. The average transition time varied between 6–8 min, as reported in a separate manuscript currently under review (Agostinelli et al. 2024). These transition times accurately reflect the NFPA standard 1710 goal dispatch response time of ~ 5–9 min (National Fire Protection Association, 2020). Upon entering the chamber participants completed a series of repeated lift and carry movements. A Rogue Fitness® sandbag (Rogue Fitness, Columbus, OH, USA) was loaded to either 61.2 kg or 38.6 kg. A deadlift 3RM > 79.4 kg and deadlift to body weight ratio > 1.25 were used as criteria for the use of the 61.2 kg sandbag; all others used the 38.6 kg sandbag. The decision to have two separate loads based on strength level but not individual relative workloads was guided in accordance with suggestions from the local fire department based on realistic expectations of their firefighters. The circuit involved 10 repeated sandbag deadlifts followed by lifting and carrying an 18.1-kg Rogue Fitness^®^ sandbag (mimicking a firehose) on a treadmill for 0.24 km. These movements were completed in a circuit fashion twice, before stopping to complete the WCST. Participants then completed two additional rounds to conclude the OTA. Participants were encouraged to complete the OTA to the best of their ability while doing so as quickly and safely as possible. Average time to complete the OTA was between ~ 19–20 min and was not significantly different by condition (Agostinelli et al. 2024). The tasks chosen in this assessment were after discussion with the local fire department and based on firefighter occupational demands outlined in previous research (Gledhill & Jamnik 1992).

### HRV analysis

HRV RR intervals were measured continuously during the WCST portion of the OTA using the Equivital EQ02 + LifeMonitor (Equivital EQ02, Hidalgo, UK) 2-lead electrocardiogram (ECG). The 256 Hz sampling frequency ensured measures within 3.9 ms for each RR value were recorded. Sampling rates greater than 250 Hz have been shown to be acceptable for HRV time-domain and frequency-domain analysis (Kwon et al. 2018). RR intervals obtained from the EquivitalEQ02 + LifeMonitor have been previously validated for HRV analysis (Akintola et al. 2016). Our analysis included time- and frequency-domain HRV measures. The time-domain measures included the standard deviation of NN intervals (SDNN; NN intervals: normal RR intervals free of artifacts), root mean square of successive differences (RMSSD), and percent of detected RR intervals greater than 50 ms different from the immediately preceding NN intervals (pNN50). Our frequency-domain measures included low-frequency (LF; 0.04–0.15 Hz) and high-frequency (HF; 0.15–0.40 Hz) power as well as the low-frequency to high-frequency contribution ratio (LF/HF ratio). These measures were chosen based on their relevance and validity during short-term HRV measurement (4–6 min) (Baek et al. 2015; Cardiology & Electrophysiology, 1996; Salahuddin et al. 2007; Shaffer & Ginsberg 2017; F. Shaffer et al. 2014a, b). Raw RR interval recordings were processed through Kubios HRV software (Kubios Oy; Kuopio, Finland).

### Statistical analysis

The Shapiro–Wilk’s test, Q–Q plot inspection, and Mauchly’s test were used to evaluate normality and sphericity, respectively. First, we conducted a repeated-measures ANOVA comparing perseverative errors by pre-OTA condition (aerobic, resistance, and control). Baseline WCST and HRV outcomes were not statistically compared to the experimental trial conditions as it was only used as a familiarization with the assessment due to the known practice effect of the assessment (Basso et al. 2001). HRV was assessed to investigate the physiological mechanism (cardiac autonomic function) behind cognitive performance. Data were assessed for outliers, which were defined as a data point greater than 3 standard deviations outside the mean. One participant’s low-frequency power, high-frequency power, and pNN50 values were classified as outliers and therefore their data were removed. A repeated-measures ANOVA was performed on HRV values during the WCST portion of the assessment to determine differences across pre-OTA conditions (aerobic, resistance, and control). For significant main effects detected, post hoc comparisons with Bonferroni adjustments were utilized for multiple comparisons. If sphericity was violated, statistical interpretations were made utilizing Greenhouse–Geisser adjusted degrees of freedom. Data are expressed as mean ± standard deviation. Significance in this investigation was set a priori at a p-value ≤ 0.05 for all measurements. R Statistical Programming Software Version 4.1.2 (RStudio; Boston, MA) was used for all statistical analyses (RStudio Team 2020). GraphPad Prism Version 9.2.0 (GraphPad Software, Boston, MA) was used to generate all figures. The application, G*Power, was utilized a priori to determine a sample size of 28 total participants necessary for a sufficiently powered study. The following values were used to estimate sample size: test family = F tests, repeated-measures ANOVA, one group, three measurements, correlation among repeated-measures = 0.5, non-sphericity correction ε = 1.0, α = 0.05, 1-β = 0.80, η2p = 0.06). The primary aim of the larger study was changes in time to complete occupational tasks, therefore the effect size was determined based on previous research related to this aim; outcomes of these studies yielded effect sizes ranging from 0.01 to 0.56 (Dennison et al. 2012; Mason et al. 2023).

## Results

### Demographics

Twenty-nine participants (female/male: 13/16) were included in the final analysis (24.90 ± 4.19 yrs; 174.47 ± 9.46 cm; 76.47 ± 12.17 kg). Average VO_2_peak was 46.2 ± 7.4 mL/kg/min and average lactate threshold at 64.5 ± 12.8% of VO_2_peak. The average total errors, perseverative errors, non-perseverative errors, and response time from the baseline WCST can be found in Table 3.

**Table 3:** Baseline Wisconsin card sorting task results

Variable	Mean ± SD
Total errors	10.97 ± 5.16
Perseverative errors	7.03 ± 2.18
Non-perseverative errors	3.93 ± 3.31
Response time (ms)	1811 ± 616

*ms* milliseconds

### Wisconsin card sorting task

**Fig. 2:**
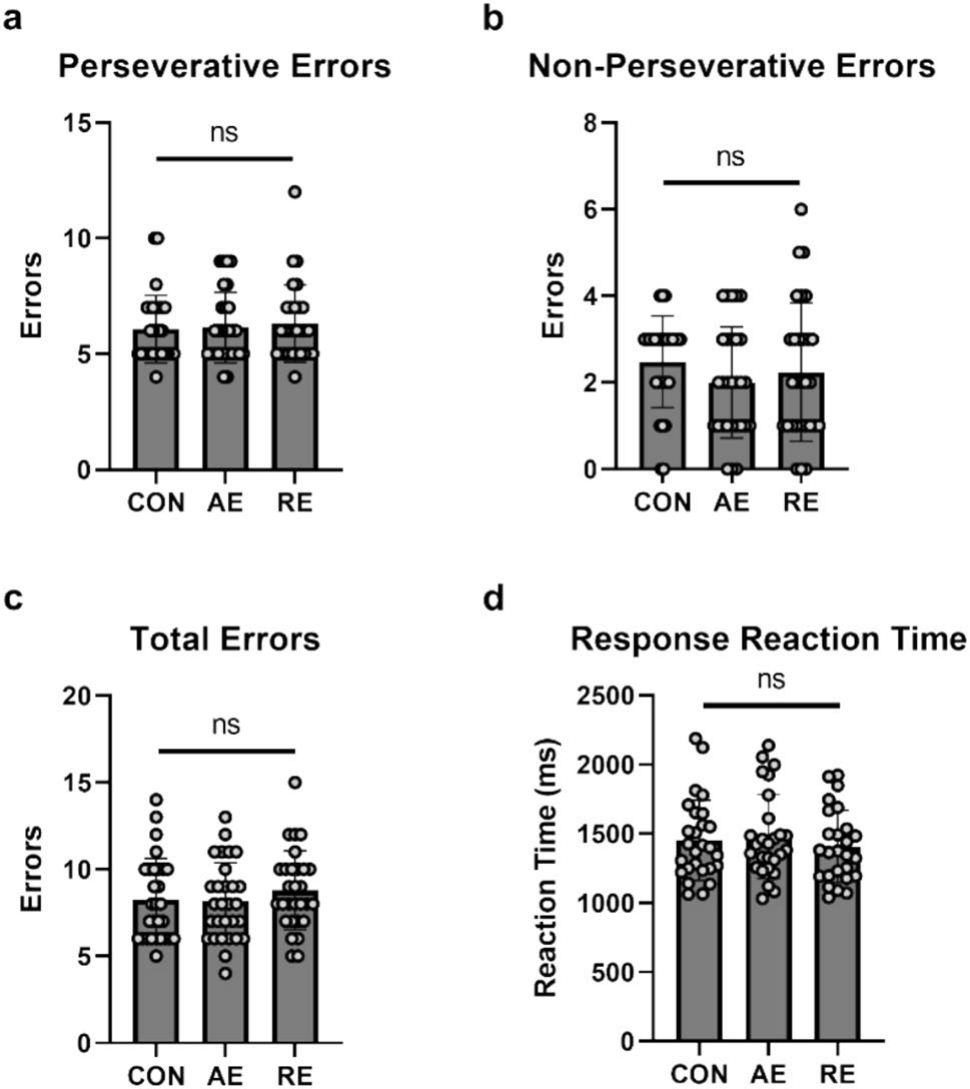
Wisconsin Card Sorting Task (WCST) outcomes; data are reported as mean ± standard deviation with individual data points overlaid; ns: not significant (p > 0.05)

Total errors were not significantly different by condition (F(2, 56) = 0.810; p = 0.45; η2p = 0.028). Perseverative errors were not significantly different by condition (F(2, 56) = 0.247; p = 0.782; η2p = 0.009). Non-perseverative errors were not significantly different by condition (F2, 56) = 0.947; p = 0.394; η2p = 0.033). Average response time did not significantly differ by condition (F(2, 46) = 2.288; p = 0.113; η2p = 0.090). Total time to complete the WCST was not significantly different by condition (F(2, 56) = 1.294; p = 0.282; η2p = 0.044). Visual representation of mean and standard deviation of WCST errors can be found in Fig. 2**.**

### Heart rate variability

**Fig. 3:**
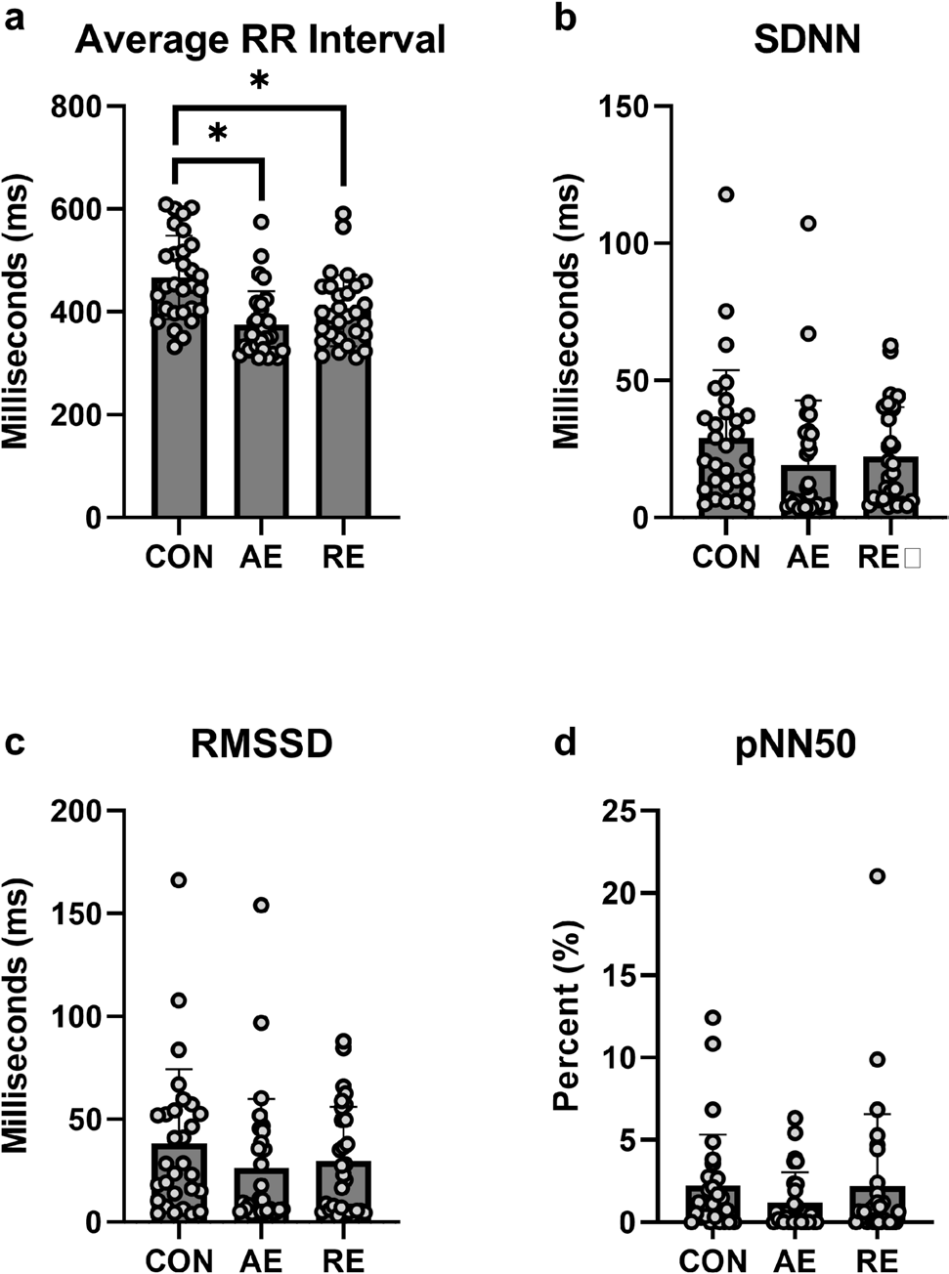
Average RR intervals and time-domain HRV outcomes; data are reported as mean ± standard deviation with individual data points overlaid. SDNN: standard deviation of NN intervals (normal RR intervals free of artifact); RMSSD: root mean square of successive RR interval differences; pNN50: percentage of successive RR intervals that differ by more than 50 ms; *denotes p < 0.05

Average RR intervals were significantly different by condition (F(2, 56) = 17.059; p < 0.001; η2p = 0.379). Post hoc analysis indicate CON Mean RR intervals were significantly higher than AE and RE conditions (ps < 0.004), but AE and RE were not significantly different (0.200). A visual representation of the mean RR intervals by condition can be found in Fig. 3.

#### Time domain analysis

SDNN did not significantly differ by condition (F(2, 58) = 3.075; p = 0.054; η2p = 0.096). RMSSD were not significantly different by condition (F2, 58) = 2.133; p = 0.128; η2p = 0.069). Participants’ pNN50 was not significantly different by condition (F(1.51, 40.88) = 0.793; p = 0.443; η2p = 0.028). Visual representation of mean and standard deviation for time-domain variables can be found in Fig. 3**.**

#### Frequency domain analysis

**Fig. 4:**
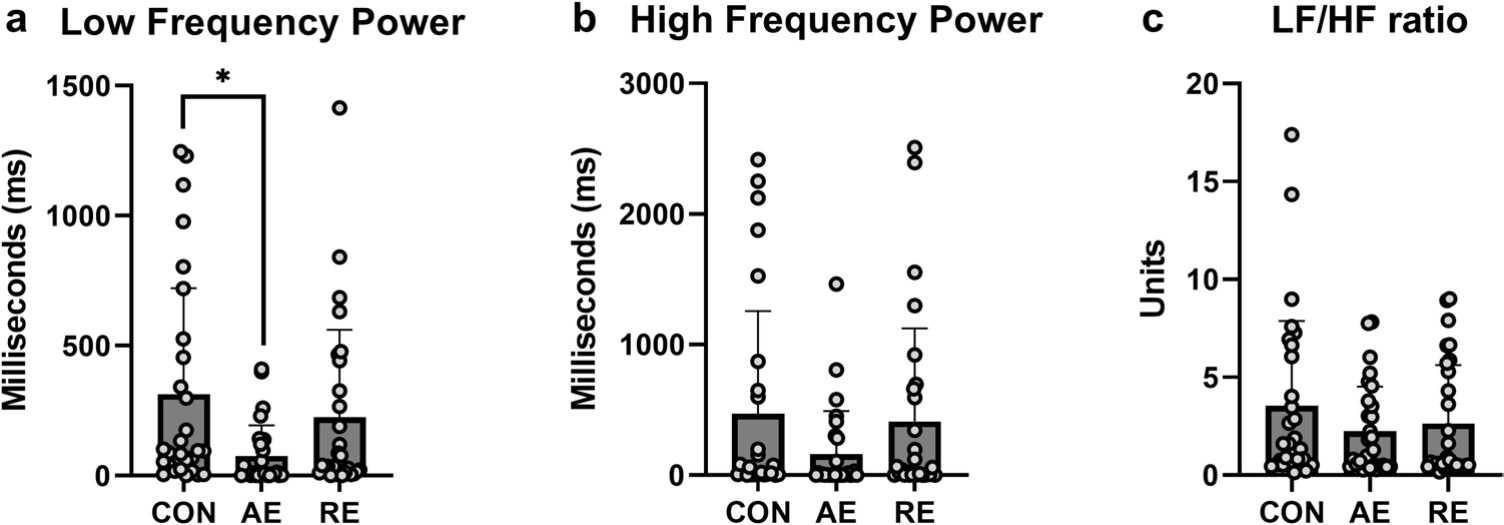
Frequency-domain HRV outcomes, data are expressed as mean ± standard deviation with individual data points overlaid. LF/HF ratio: low-frequency to high-frequency power ratio; *denotes p < 0.05

LF was significantly different by condition (F(1.57, 42.33) = 4.313; p = 0.028; η2p = 0.138). Post hoc analysis indicated that LF was significantly lower after AE when compared to control CON (p = 0.014). LF was not significantly different following RE when compared to CON or AE (ps > 0.085). HF was not significantly different by condition (1.44, 39) = 1.938; p = 0.167; η2p = 0.067). LF/HF ratio did not differ significantly by condition (F(1.65, 44.43) = 1.476; p = 0.239; η2p = 0.052). Visual representation of mean and standard deviation for frequency-domain variables can be found in Fig. 4.

## Discussion

This study is the first to our knowledge to look at the influence of acute exercise on cognitive function during simulated firefighter occupational tasks, and its relation to HRV metrics associated with autonomic control. Our main findings suggest that there was not evidence that cognitive flexibility in a simulated occupational setting, as assessed by the WCST during the OTA, was impacted by prior acute exercise. Prior acute exercise (resistance and aerobic) did result in a significant decrease in mean RR interval during the cognitive assessment. This suggests that prior acute exercise resulted in an elevated heart rate during the cognitive assessment when compared to the control condition. Additionally, acute exercise only resulted in small, non-significant effects on time-domain HRV variables. Investigation of frequency-domain variables revealed high-frequency power and the low-frequency to high-frequency contribution ratio were minimally, and not significantly, different during the cognitive assessment based on condition. However, low-frequency power, which is associated with parasympathetic tone, was significantly lower during the cognitive assessment following aerobic exercise. Therefore, aerobic exercise before an emergency call may influence metrics of parasympathetic tone, during cognitively demanding occupational situations.

Contrary to our hypothesis, we observed negligible, non-significant differences in cognitive flexibility during simulated firefighter occupational tasks regardless of prior acute exercise (resistance or aerobic). Previous research has not looked at changes in cognitive flexibility while firefighting, but did observe differences in other domains of cognitive function during firefighting tasks (Morley et al. 2012; Zare et al. 2018). One possible explanation for the lack of change could be that the occupational task demands chosen for the OTA may have required less cognitive resources than other firefighting tasks. For example, breaching through a door or navigating debris and physical obstacles may require more cognitive recourses than the lift and carry tasks chosen in this investigation. If cognitive resources are drained from more complex occupational tasks, subsequent cognitive performance may be more sensitive to the effects of acute exercise (Head et al. 2016; Hutchinson & Tenenbaum 2007; Slimani et al. 2018).

The findings did not provide evidence supporting our original hypothesis that prior resistance exercise would impair cognitive flexibility in a simulated occupational setting, in part due to a reduction in metrics of HRV associated with parasympathetic tone. Our findings suggest that despite prior aerobic exercise resulting in reductions in some HRV metrics (low-frequency power) that are associated with both parasympathetic and sympathetic activity, there were negligible differences in cognitive flexibility. Taking a closer look at our frequency metric of HRV as a whole may provide more insight into the lack of significant change. Low-frequency power is often associated with parasympathetic, during short-term monitoring when seated and upright, but sympathetic activity, when in stressful situations or with recent exercise or activity (Axelrod et al. 2008; Fred Shaffer et al. 2014a, b) while high-frequency power, is almost exclusively associated with parasympathetic activity (Cardiology & Electrophysiology, 1996). While there was not a significant decrease in high-frequency power following aerobic exercise (mean difference from control = 307.10; mean differences from resistance exercise 551.15; p = 0.067), there was a large numeric decrease when compared to the control session. Additionally, prior acute exercise resulted in small, non-significant changes in LF/HF ratio, which is suggested to provide insight into the balance of parasympathetic and sympathetic activity (Fred Shaffer et al. 2014a, b). Since low-frequency power was monitored for a short duration in a seated, upright condition, but immediately after exertional tasks in a hot, humid environment the complexity of the setting may explain the lack of changes in cognitive outcomes as there may have a balance of parasympathetic and sympathetic activities allowing for a maintenance of cognitive flexibility, which may be further supported by the lack of difference in LF/HF ratio between pre-OTA exercise conditions (AE, RE, CON).

Average RR interval time during the cognitive assessment significantly decreased by condition, confirming that heart rate was in fact higher during the cognitive assessment following conditions that involved exercise; however, there was no evidence of a difference between the aerobic and resistance exercise conditions. Acute exercise resulted in small, non-significant effects on time-domain metrics of HRV during the WCST in a simulated occupational setting when compared to the control condition. Acute exercise resulted in small, non-significant effect on SDNN and RMSSD compared to control. These time-domain variables are indicators of parasympathetic activity (Shaffer & Ginsberg 2017). This may suggest that acute exercise (resistance or aerobic) prior to firefighter occupational tasks may not impair parasympathetic activity, which is responsible for regulating blood pressure and heart rate (Benarroch 2014). This is an important consideration when considering that these variables play an integral role in cognitive function and decision-making (Böhm et al. 2015).

Low-frequency power is often associated with parasympathetic and sympathetic activity, while high-frequency power reflects solely parasympathetic cardiac drive (Axelrod et al. 2008; Berntson et al. 1997; Fred Shaffer et al. 2014a, b). Therefore, depending on the conditions LF/HF ratio is thought to measure sympathetic/parasympathetic balance. It is worth noting that this has been challenged in recent work and therefore these findings should be taken with caution (Billman 2013; Von Rosenberg et al. 2017). Previous research suggests that when low-frequency power is decreased there is an increase in blood pressure variability, an impaired ability to respond to acute challenges to the maintenance of BP, and an increased risk of sudden cardiac events (Goldstein et al. 2011). If this impairment continues throughout occupational scenarios, this could suggest that acute on-shift aerobic exercise may increase risk for sudden cardiac events (Soteriades et al. 2011). This risk is especially important when considering the large proportion of firefighters with existing co-morbidities for cardiac disease (Gendron et al. 2018) and their high rates of sudden cardiac deaths (Smith et al. 2013). These deaths consist of over 45% of all duty-related fatalities. This risk should be considered when recommending on-shift exercise for firefighters. Resistance training exercise may provide a safer option for on-shift exercise, while aerobic exercise is completed off shift. This recommendation, however, should be taken with caution to assure that aerobic exercise is not neglected as it is a vital component of both health and performance in firefighters (Chizewski et al. 2021).

The differential effects seen across the literature could be related to exercise prescription variables such as volume and intensity varying between studies on acute exercise and cognitive function. Generally, positive effects are most prominent following acute aerobic exercise, but other literature has found no changes in cognition in agreeance with the results of our study (Gothe et al. 2013; Hogan et al. 2013; Jäger et al. 2014; Piepmeier et al. 2015; Soga et al. 2015; Weng et al. 2015). However, these prescription choices may be attributable to differences from previous research. We chose HIIT for its strong translation to the occupational demands of firefighters as well as considering the time effectiveness of it for practical implementation in fire services. Previous research commonly uses light to moderate continuous steady-state exercise (Chang et al. 2012) compared to the high-intensity interval training used in the current investigation. Research on the ideal intensity is still inconclusive as some research suggests the biggest effects are seen following high-intensity exercise (Barenberg et al. 2015; Córdova et al. 2009; Pindus et al. 2019). However, others suggest moderate intensity results in the largest enhancements in cognition (Brisswalter et al. 2002; McMorris & Hale 2012). Similarly, even though research in the past looking at the acute cognitive effect of resistance exercise commonly prescribed training with higher loads, lower set counts, and more total exercises (Huang et al. 2022), our exercise prescriptions were used to more accurately reflect the strength demands and time restriction of firefighters. This may suggest intensity or volume plays a large role in the enhancement of cognitive flexibility after aerobic and resistance exercise.

These exercise prescription differences between the current investigation and previous research may also influence our HRV metrics. One study that guided our hypothesis was an investigation into acute resistance and aerobic exercise’s influence on HRV during recovery. This previous research investigated the notion that resistance exercise caused a greater decline in HRV metrics, including low- and high-frequency power, that was still present during recovery (Heffernan et al. 2006). Their findings suggested that resistance exercise may result in a more dramatic decrease in parasympathetic tone compared to aerobic exercise due to different neural modulation following each mode of exercise. However, the previous study’s resistance exercise prescription included more exercises at a higher training volume, lower intensity, and with shorter rest periods, compared to our prescription. Additionally, this study used a steady-state cycling at 65% of VO_2_peak aerobic session, compared to our study which used alternating high (90% HRR) and low (50% HRR) intervals treadmill running. This interval structure may induce parasympathetic and sympathetic responses more akin to resistance exercise rather than steady-state aerobic exercise. Therefore, future work should consider investigating the impact of different exercise prescription variables and their impact on ensuing cognitive performance during firefighter occupational tasks.

In addition to the prescription considerations, a potential factor that explains differences in our findings from previous literature is highlighted in a 2012 meta-analysis which analyzed the time course effect of acute exercise on cognitive function. The summative data from the literature to determine the differences in cognitive performance changes both immediately after exercise and most prominently after 11–20 min post-exercise (Chang et al. 2012). During our investigation, the methods of exercise training were done approximately 18 min before the WCST, however the time between exercise and cognition included physically exerting occupational tasks that may have influenced this response. Additionally, the occupational tasks were completed as fast as possible by participants; therefore, there was variability in the time (~ 8–23 min) between the end of exercise and the start of the WCST.

Previous work in heart rate variability research highlights a limitation of this investigation, which is that cognitive function and HRV measures are complex and difficult to attribute to a single physiological response (Diamond 2013; Linder et al. 2023; Pham et al. 2021). Therefore, interpretations from these outcomes should be taken with caution. The variability of these measures along with the fact that our sample size was determined based on a separate aim of the larger study, it is possible that a larger sample size would improve the ability to detect differences in these outcomes. Additionally, there was not a baseline cognitive assessment prior to each experimental condition or more frequently throughout the OTA (i.e., pre- and post-assessment). This was an intentional decision to provide timely transitions between exercise, OTA, and recovery that would more accurately reflect the experience of a firefighter. Future research should consider the addition of assessments to understand the day-to-day variability and time course of cognitive performance in firefighters following acute exercise. The aim of this investigation was to assess the relation between HRV and cognitive flexibility during simulated firefighting after acute exercise, however there are many other areas of physiological changes such as lactate concentrations (Xue et al. 2022), blood flow (Leeuwis et al. 2018), and core temperature (Ashworth et al. 2021; Jung et al. 2022) after exercise that may contribute to cognitive performance. It is important to note that while our replication of relevant environmental conditions (Agostinelli et al. 2023; Aisbett et al. 2012; Williams-Bell et al. 2017) and tasks (Gledhill & Jamnik 1992) using previous literature and the guidance of the local fire department the simulated task assessment is still likely to vary from real-world scenarios. Specifically, it is impossible to create the level of stress experienced during a real emergency in a simulated environment which could impact HRV responses. Additionally, future research in this area should consider other domains of cognition (Harvey 2019; Kiely 2014; Lezama et al. 2004; Miyake et al. 2000) to help further elucidate the impacts of acute exercise on cognitive function during firefighter occupational tasks. Lastly, it is worth acknowledging that the current investigation we chose to use local community members rather than career firefighters to ensure a larger sample size was obtained, due to the limited number of local firefighters. Previous literature has highlighted that long-term career firefighters may have protective adaptations from heat which could influence the responses investigated in the current study (Wright et al. 2013).

## Conclusions

The results of this investigation suggest that firefighters who perform acute aerobic or resistance exercise while on shift may not experience deleterious effects on cognitive flexibility. However, metrics of HRV may be depressed by prior performance of acute aerobic exercise, which may influence heart rate and blood pressure regulation. It is important to acknowledge that this is not a recommendation to avoid aerobic exercise, as it is vital to cardiovascular health and occupational safety in firefighters (Jeung et al. 2022). Instead, firefighters should be aware of this potential risk when scheduling aerobic exercise around their work schedule, such as completing aerobic training when off duty.

## Data Availability

Data for this project is available on figshare 10.6084/m9.figshare.27411336.

## Abbreviations

HRV: Heart rate variability
PAR-Q: Physical activity readiness questionnaire
RE: Resistance exercise
AE: Aerobic (high-intensity interval) exercise
CON: Rested control session
OTA: Occupational task assessment
HRR: Heart rate reserve
3RM: Three-repetition maximal lift
VO_2_peak: Peak oxygen consumption
WCST: Wisconsin card SORTING Task
USG: Urine specific gravity
NSCA: National strength and conditioning association
HIIT: High-intensity interval training
ECG: Electrocardiogram
SDNN: Standard deviation of NN intervals
RMSSD: Root mean square of successive differences
pNN50: Percent detected RR intervals > 50 ms different from the immediately preceding NN interval
LF: Low-frequency power
HF: High-frequency power
LF/HF: Low frequency/high frequency ratio
ANOVA: Analysis of variance
